# The role of mitochondrial dynamics in metabolic dysfunction–associated steatotic liver disease

**DOI:** 10.3389/fmed.2026.1804561

**Published:** 2026-05-13

**Authors:** Ningxi Yang, Yarong Hao

**Affiliations:** Department of Geriatrics, Wuhan University Renmin Hospital, Wuhan, China

**Keywords:** metabolic dysfunction–associated steatotic liver disease, mitochondrial dynamics, mitochondrial fusion and fission, mitophagy, therapeutic strategies

## Abstract

Metabolic dysfunction-associated steatotic liver disease (MASLD) encompasses a spectrum of manifestations arising from multisystem metabolic dysfunction impacting the liver. This spectrum includes hepatic conditions such as steatohepatitis, liver fibrosis, cirrhosis, and hepatic malignancies, as well as extrahepatic conditions like type 2 diabetes, atherosclerotic diseases, and chronic kidney diseases. MASLD has emerged as one of the most prevalent chronic liver diseases worldwide, characterized by a complex pathogenesis and a current lack of effective pharmacological interventions. With societal development and improved living standards, the incidence of MASLD is projected to rise, necessitating urgent research into its pathogenesis and the development of effective prevention and treatment strategies. Mitochondria, as the intracellular hubs of energy metabolism, are crucial in the onset and progression of MASLD through their dynamic processes. In recent years, the role of mitochondrial dynamics in the pathogenesis of MASLD has garnered increasing scholarly attention. This article provides a comprehensive overview of recent advancements in the relationship between mitochondrial dynamics and MASLD. It delves into the molecular mechanisms underlying mitochondrial fusion and fission, examines the functional abnormalities of mitochondrial dynamics in MASLD, and explores potential therapeutic strategies. These studies aim to offer novel insights and methodologies for the prevention and treatment of MASLD.

## Introduction

Non-alcoholic fatty liver disease (NAFLD) is a clinicopathological syndrome characterized by the excessive accumulation of fat in liver cells, independent of alcohol consumption or other identifiable liver injury factors. This condition progresses through a continuum of pathological stages, ranging from early fat deposition, known as simple fatty liver (NAFL), to non-alcoholic steatohepatitis (NASH), liver cirrhosis, and potentially hepatocellular carcinoma (1). In 2023, the Multi-Society Delphi Consensus on the New Naming of Fatty Liver Disease proposed renaming NAFLD to “Metabolic Dysfunction-Associated Steatotic Liver Disease” (MASLD), abbreviated as metabolism-related fatty liver disease (2). The revised nomenclature underscores the critical role of metabolic disorders in both the identification and treatment of diseases. Currently, therapeutic strategies for MASLD remain ineffective, and its pathophysiological mechanisms are not yet fully elucidated. It is crucial to distinguish between simple hepatic steatosis and steatohepatitis, as they represent distinct pathological stages within the MASLD spectrum. Simple steatosis is characterized by isolated lipid accumulation in hepatocytes without significant inflammation or cellular injury, whereas steatohepatitis features additional hepatocyte ballooning, lobular inflammation, and variable fibrosis, which confers a higher risk of progression to cirrhosis and hepatocellular carcinoma. While steatosis may remain stable or even regress, steatohepatitis is the key driver of liver-related morbidity and mortality, necessitating targeted therapeutic intervention.

MASLD is closely intertwined with systemic metabolic disorders. Epidemiological studies have shown that up to 70–80% of patients with type 2 diabetes mellitus (T2DM) and 50–60% of individuals with obesity have coexisting MASLD (3). Conversely, the presence of MASLD significantly increases the risk of developing T2DM, hypertension, and atherosclerotic cardiovascular diseases, independent of traditional risk factors (4). This bidirectional relationship underscores that MASLD is not merely a liver condition but a hepatic manifestation of systemic metabolic dysfunction.

Mitochondria are double-membrane organelles that serve as the primary sites for ATP production, calcium buffering, and apoptotic regulation, and they form a highly dynamic network within cells. Their proper function depends on a delicate balance between fusion and fission events, collectively termed mitochondrial dynamics. Mitochondrial dynamics encompass a series of intracellular changes—fusion, fission, and degradation—that are essential for maintaining mitochondrial morphology, function, and quantity, and form the foundation for mitochondrial quality control (5). The equilibrium between fusion and fission is vital for mitochondrial function and quality control, as it ensures efficient material transport, enhances mitochondrial homogeneity, and regulates the oxidative phosphorylation process (6). The regulation of mitochondrial dynamics facilitates mitochondrial quality control by modulating mitochondrial morphology, material exchange, mitochondrial DNA (mtDNA) inheritance, and the segregation and degradation of damaged mitochondria (7, 8). The mitochondrial quality control (MQC) mechanism encompasses a diverse array of strategies that have evolved in organisms to monitor and preserve mitochondrial integrity. In instances where mitochondria incur damage or exhibit abnormal functionality, these mechanisms activate specific repair or clearance processes, including mitochondrial dynamics, repair of mitochondrial genetic material, the mitochondrial unfolded protein response (UPRmt), mitophagy, and mitochondrial biogenesis (9–11). The UPRmt is a retrograde stress signaling pathway that upregulates chaperones and proteases to restore protein homeostasis within the mitochondrial matrix, and it is closely linked to metabolic diseases such as MASLD (9, 10). Collectively, these measures ensure the maintenance of mitochondrial homeostasis, structure, quantity, and functionality, thereby supporting the normal operation of cells and the entire organism (12). Notably, mitochondrial dynamics play a particularly vital role in safeguarding and facilitating the biological functions of mitochondria. Within living cells, mitochondria undergo continuous cycles of fusion and fission, altering their morphology, size, and position to adapt to stressors such as hypoxia, pressure overload, and inflammation. These morphological changes are intricately linked to functional adaptation, underscoring the central role of mitochondria in maintaining cellular homeostasis.

Mitochondrial dysfunction in the liver is potentially the primary etiological factor of the disease, evidenced by abnormal mitochondrial morphology, mtDNA damage, metabolic disorders, oxidative stress, autophagy abnormalities, and other associated factors (13). Advanced research into mitochondrial dynamic behavior has highlighted that disruptions in mitochondrial dynamics significantly contribute to the onset and progression of MASLD, suggesting that maintaining mitochondrial homeostasis may be pivotal for therapeutic strategies.

## Interplay between mitochondrial dynamics and mitochondrial quality control in MASLD

Mitochondrial quality control (MQC) is a multidimensional system that preserves mitochondrial homeostasis through the coordinated actions of dynamics (fusion/fission), mitophagy, biogenesis, and repair of mitochondrial DNA (mtDNA) (5, 12). Importantly, mitochondrial dynamics and MQC are not independent processes; rather, they are tightly coupled. Mitochondrial fusion facilitates the mixing of contents, allowing functional complementation and dilution of damaged proteins and mtDNA, thereby serving as a first-line quality control mechanism that can rescue mildly impaired mitochondria (14). Conversely, when damage exceeds the capacity of fusion-mediated repair, fission acts to segregate dysfunctional mitochondrial subdomains, producing smaller fragments that are targeted for mitophagy (15, 16). Thus, the balance between fusion and fission determines whether a damaged mitochondrion is repaired or eliminated.

In MASLD, this coupling is disrupted. Chronic metabolic stress (e.g., hyperglycemia, free fatty acids) impairs fusion protein expression (Mfn1, Mfn2, Opa1) and upregulates fission proteins (Drp1, Fis1), tilting the balance toward excessive fission (17, 18). The resulting fragmented mitochondria are more prone to mitophagy, but sustained overload may overwhelm autophagic clearance, leading to accumulation of dysfunctional mitochondria, increased reactive oxygen species (ROS), and mtDNA release (16, 19). Moreover, impaired fusion compromises the efficiency of MQC by limiting complementation, allowing damaged components to persist and propagate cellular injury. This vicious cycle accelerates the progression from simple steatosis to steatohepatitis and fibrosis (20).

Understanding the crosstalk between dynamics and MQC has therapeutic implications. Interventions that restore fusion (e.g., Mfn2 upregulation) or inhibit excessive fission (e.g., Mdivi-1) not only balance dynamics but also enhance mitophagy and mitochondrial biogenesis, thereby improving overall MQC (18, 21). Consequently, targeting the dynamics-MQC axis represents a promising strategy for MASLD treatment.

## Mitochondrial energy production and its regulation by dynamics in MASLD

Mitochondria are the primary cellular sites for ATP generation through oxidative phosphorylation (OXPHOS), which couples electron transport chain (ETC) activity with ATP synthesis. Proper mitochondrial architecture—including cristae density and network connectivity—is essential for maintaining high OXPHOS efficiency (13, 22). Mitochondrial dynamics directly influence energy production. Fusion preserves cristae integrity and maximizes the surface area for ETC complexes, thereby sustaining ATP output under physiological conditions (14, 23). Conversely, fission allows redistribution of mitochondrial mass to subcellular regions with high energy demands (e.g., sites of active lipid metabolism) and facilitates turnover of damaged ETC components (15, 16). The balance between fusion and fission thus determines the spatial and temporal distribution of ATP-generating capacity within cells.

In MASLD, this balance is disturbed. Excessive fission and impaired fusion lead to mitochondrial fragmentation, reduced cristae density, and loss of membrane potential (ΔΨm), all of which compromise OXPHOS (22, 24). Studies in high-fat diet–fed rodents and NASH patients have shown decreased activity of complexes I, III, and V, along with reduced ATP levels in hepatocytes (24, 25). Impaired fatty acid *β*-oxidation—the primary substrate for hepatic ATP production during fasting—further exacerbates energy deficit, promoting lipid accumulation and insulin resistance (23). Moreover, fragmented mitochondria produce more reactive oxygen species (ROS) and release mtDNA, triggering inflammatory cascades that worsen steatohepatitis (16, 19). Thus, we suggest that restoring balanced mitochondrial dynamics may improve energy homeostasis and alleviate MASLD progression.

## Abnormal mitochondrial dynamics in MASLD

The quantity and morphology of mitochondria are indicative of the metabolic and functional status of cells (5, 14). Typically, cells exhibiting high metabolic activity possess a greater number of mitochondria. For example, hepatocytes with elevated metabolic rates contain numerous mitochondria with well-developed cristae, serving as essential metabolic and signaling centers that facilitate normal liver metabolic functions, adaptability of liver cell functions, and cellular survival.

Mitochondrial dysfunction, characterized by excessive peroxide production, pro-inflammatory factor activation, and ultrastructural damage, can worsen intracellular lipid peroxidation, significantly harm the liver, and exacerbate non-alcoholic steatohepatitis (26). While the link between mitochondrial dynamics and MASLD is not fully elucidated, recent research indicates potential mitochondrial dynamics disorders in MASLD. Studies have demonstrated a strong association between mitochondrial dysfunction and both hyperglycemia and insulin signaling pathway disruptions, leading to reduced energy production in the mitochondrial oxidative respiratory chain (24, 27). This energy deficit is compensated by downregulating fusion protein expression and upregulating fission protein expression (17, 18, 23).

### Cell type-specific considerations in mitochondrial dynamics

While most studies on MASLD have focused on hepatocytes, which are the primary site of lipid accumulation and metabolic dysfunction (5, 12), other liver cell types also exhibit distinct mitochondrial dynamics and may contribute to disease progression. Kupffer cells (resident liver macrophages) show altered mitochondrial fission/fusion balance upon lipopolysaccharide or free fatty acid stimulation, leading to inflammasome activation and cytokine release that aggravate steatohepatitis (7, 8). Hepatic stellate cells (HSCs) undergo activation during fibrosis, and mitochondrial fission has been implicated in their transdifferentiation into myofibroblasts, promoting extracellular matrix deposition (10, 13). Cholangiocytes (bile duct epithelial cells) also display mitochondrial adaptations in response to cholestatic injury, though their role in MASLD remains less defined (11). Thus, future investigations should delineate cell-type-specific mitochondrial dynamics to fully understand MASLD pathogenesis and identify targeted therapeutic approaches.

### Abnormalities in mitochondrial structure and function in MASLD

*In vitro* experiments reveal that high-glucose environments impact the morphology and function of liver cell mitochondria (22). Abnormal mitochondrial morphology and functional disorders are frequently observed in the liver tissues of MASLD patients. Mitochondrial structural abnormalities are a critical factor in the progression from simple steatosis to steatohepatitis, characterized by significant alterations in mitochondrial morphology, such as swelling of hepatic mitochondria, fragmentation of cristae, stacking of crystalline inclusions, and the emergence of giant mitochondria, among other changes (28). These mitochondrial defects result in increased permeability of the inner mitochondrial membrane, leading to disruptions in intracellular homeostasis. The excessive accumulation of dysfunctional mitochondria within cells can significantly impair cellular metabolism and potentially trigger cell death.

### Dyskinetic mitochondrial energy metabolism abnormality

As the “powerhouses” of cells, mitochondria are integral to energy conversion processes. Studies suggest that the dynamic balance between mitochondrial fusion and fission is maintained through a protective mechanism, which is vital for preserving the normal morphology, structure, and function of mitochondria. When subjected to adverse conditions such as oxidative stress and pressure, the equilibrium of mitochondrial dynamics can be disrupted, resulting in damage to the network structure and subsequent functional disorders and abnormal energy metabolism. This disruption ultimately impairs liver cell function and facilitates the progression of MASLD. Mitochondrial fusion plays a crucial role in repairing mildly damaged cellular components, while mitochondrial fission not only increases the mitochondrial count but also facilitates the removal of damaged mitochondria via mitophagy. Furthermore, the remodeling of mitochondrial cristae is intricately linked to the pathogenesis of MASLD. Mitofusin 1 (Mfn1) is involved in regulating the reconstruction of mitochondrial cristae morphology. Alterations in the number and morphology of mitochondrial cristae can impact the normal functioning of the mitochondrial respiratory chain, thereby influencing the progression of fatty liver disease. When fission proteins are excessively activated or inhibited, the equilibrium of oxidative phosphorylation is disrupted, leading to a weakening of the cellular respiratory chain oxidative phosphorylation (OXPHOS). This disruption can result in the accumulation of reactive oxygen species, a reduction in mitochondrial membrane potential (MMP), and alterations in the expression of mtDNA, all of which may adversely impact cellular health.

A population-based study has demonstrated that, in cases of non-alcoholic steatohepatitis (NASH), the expression level of Mitofusin 2 (Mfn2) in skeletal muscle exhibits a downward trend compared to cases of simple hepatic steatosis, whereas the expression level of Dynamin-related protein 1 (Drp1) significantly increases (29). In an animal study, Dominguez-Perez et al. (30) investigated the expression levels of proteins related to mitochondrial dynamics in the liver cells of mice subjected to a high-cholesterol diet. The findings revealed a significant reduction in the expression levels of Mfn1, Mfn2, and Opa1, which are associated with mitochondrial fusion, while the expression level of Drp1, associated with mitochondrial fission, was significantly elevated. This evidence indicates that cholesterol intake may influence the equilibrium between mitochondrial fission and fusion, thereby adversely affecting the lipid storage capacity of hepatocytes. Furthermore, this disruption in mitochondrial dynamics could lead to modifications in the expression of respiratory chain complex proteins, resulting in structural alterations of hepatic mitochondria and impairing hepatocellular function, which may ultimately contribute to the onset of severe hepatic disorders.

### Mitochondrial dynamics imbalance in dysregulated fatty acid metabolism and increased oxidative stress

Given that mitochondria are the primary site for fatty acid oxidation, such an imbalance hinders fatty acid metabolism, impairs oxidation and glycolysis, thereby promoting hepatic lipid accumulation and exacerbating MASLD progression. Hsu et al. (31) demonstrated that leptin promotes mitochondrial fusion in murine hepatic cells, thereby mitigating lipid accumulation induced by elevated glucose levels. Additionally, leptin was shown to inhibit excessive mitochondrial fission in hepatic cells, contributing to the alleviation of steatosis. In a related study, Bourebaba et al. (23) developed an *in vitro* model of non-alcoholic fatty liver disease (NAFLD) and observed a significant reduction in mitochondrial fusion protein levels, alongside an increase in fission protein levels, which are correlated with NAFLD pathogenesis. Galloway et al. (32) provided *in vivo* evidence that attenuating mitochondrial fission can effectively ameliorate simple steatosis resulting from increased dietary fat consumption. Furthermore, Cruz Hernandez et al. (33) reported that a high-sugar diet-induced hepatic metabolic disorder is associated with a marked elevation in Drp1 levels, leading to an imbalance in mitochondrial dynamics. Collectively, these studies suggest that mitochondrial fusion may play a pivotal role in mitigating hepatic steatosis.

The mitochondrial respiratory chain serves as the primary subcellular source of ROS, which have the potential to damage mitochondrial proteins, lipids, and mitochondrial DNA, disrupt redox homeostasis, elevate inflammatory responses, and impair mitochondrial energy metabolism. NASH is intricately linked to mitochondrial damage induced by oxidative stress (24). In patients with NASH, the production of ROS due to localized inflammatory reactions in the liver increases significantly, resulting in elevated levels of peroxidized lipids, disruption of redox homeostasis, and the release of additional cytokines, thereby exacerbating mitochondrial damage and promoting cell apoptosis (25). In prevalent metabolic disorders, the oxidative capacity of hepatic mitochondria may be augmented in response to an increased lipid metabolic burden, thereby mitigating excessive lipid accumulation. An imbalance in mitochondrial dynamics may constitute a significant factor in the development of fatty liver, particularly in the context of chronic diseases with progressive trajectories, such as obesity and type 2 diabetes. This scenario increases the liver’s vulnerability to oxidative stress and exacerbates inflammatory responses or fibrosis in hepatocytes, potentially progressing to steatohepatitis, liver fibrosis, and even cirrhosis (23).

## Mitochondrial fusion and fission in metabolic fatty liver disease

### Mitochondrial fusion in metabolic fatty liver disease

**Figure 1:**
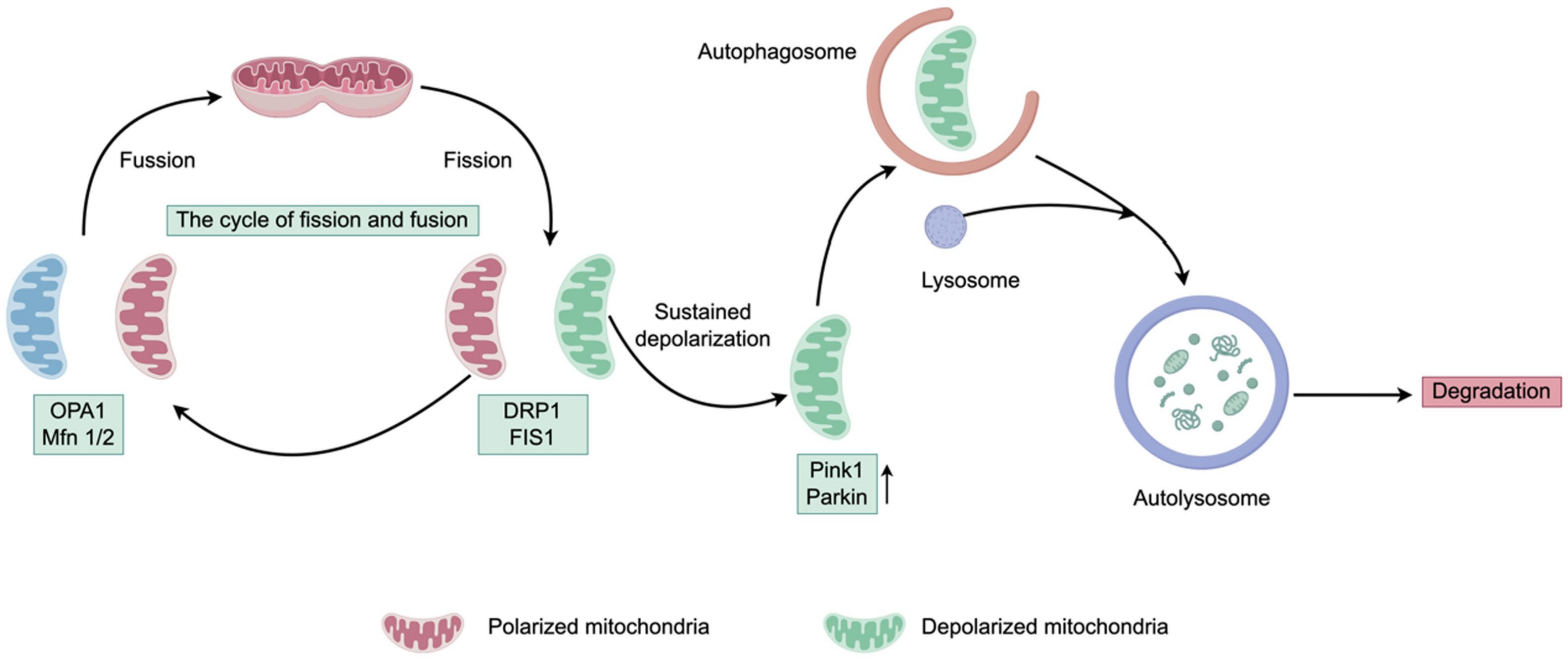
Schematic of mitochondrial fusion, fission, and mitophagy. Mitochondria are highly dynamic organelles characterized by frequent fusion and fission events. The process of mitochondrial fusion serves to mitigate the impact of mutations by amalgamating mutated mitochondrial DNA (mtDNA) with non-mutated mtDNA. In instances where mitochondrial damage surpasses a critical threshold, fission is initiated, leading to mitochondrial depolarization. Post-fission, mitochondria may either restore their membrane potential and re-enter the functional cycle or persist in a depolarized state. The fusion of mitochondria is facilitated by mitofusins (Mfns) and optic atrophy protein 1 (OPA1), whereas fission is mediated by dynamin-related proteins and mitochondrial fission 1 protein (FIS1). Persistent depolarization results in the cleavage of OPA1 and a reduction in Mfn1, accompanied by the accumulation of PINK1 and Parkin proteins. Ultimately, these mitochondria are targeted for degradation via mitophagy after residing in the pre-autophagic pool for several hours.

Mitochondrial fusion represents a fundamental morphological alteration of mitochondria. Following fusion, mitochondria elongate, their contents merge, and the adenosine triphosphate (ATP) content rises. Mitochondrial fusion is a complex biological process that involves the activation of dynamin-related GTPases, the anchoring and fusion of the outer mitochondrial membrane (OMM) and inner mitochondrial membrane (IMM), among other processes (34). In mammalian cells, the regulation of these processes relies on three guanosine triphosphatases (GTPases). At the level of the outer mitochondrial membrane, Mfn1/2 undergo GTP hydrolysis, which induces conformational changes in Mfn. This results in the formation of homodimers or heterodimers through disulfide bonds, connecting in an antiparallel manner and initiating the fusion process of adjacent mitochondrial membranes. Mfn1 and Mfn2 are critical components of mitochondrial fusion, as illustrated in Figure 1 (35). The absence or diminished expression of either protein will significantly impede mitochondrial fusion, with a dual deficiency of both proteins completely halting the fusion process and exacerbating cytological issues. Additionally, Opa1 is a crucial fusion protein, as illustrated in Figure 1. Through its distinct transcriptional and proteolytic mechanisms, Opa1 generates two active forms: long Opa1 (L-Opa1), which is anchored to the inner mitochondrial membrane to facilitate its integration, and short Opa1 (S-Opa1), which is released into the intermembrane space, contributing to inner membrane fusion and cristae remodeling (35). These two active forms of Opa1 collaborate to ensure the efficient progression of inner mitochondrial membrane fusion.

Mitochondrial fusion supports mitochondrial connectivity, material exchange, and the sharing of mtDNA and proteins, thereby promoting metabolic synergy, preventing genetic loss, and aiding in the repair of damaged mitochondria to sustain normal function (14, 36). Mitochondria form networks via membrane fusion to meet cellular metabolic demands. The inner membrane’s crista structure enhances surface area, facilitating mitochondrial communication, optimizing oxidative phosphorylation, and supporting cellular activities. Hyper-fused networks, compared to fragmented ones, are more resilient to damage and apoptotic signals, reduce ROS production, boost ATP supply, enhance material exchange, maintain structural homology, and preserve genetic integrity during cell division (37, 38). In mammals, mitochondrial fusion proteins like Mfn1, Mfn2, and Opa1 are key research subjects. Hepatic insulin resistance (IR) and steatosis are critical pathophysiological characteristics of metabolic disorders such as non-alcoholic fatty liver disease (NAFLD) and type 2 diabetes. The transcriptional regulator, peroxisome proliferator-activated receptor *γ* coactivator 1 (PGC-1), serves as the principal regulator of mitochondrial biogenesis and is a key initiator of genes associated with mitochondrial biosynthesis. It plays a pivotal role in the regulation of mitochondrial fusion-related proteins.

Research indicates that in environments characterized by hyperglycemia and hyperlipidemia, the Ca2+/PGC-1α/OPA1 axis is suppressed, resulting in decreased expression of Opa1, Mfn1, and Mfn2, thereby weakening mitochondrial fusion, as demonstrated in *in vivo* and *in vitro* models (39) and in cultured human podocytes (27). Impairments in hyperglycemic and insulin signaling conditions lead to diminished expression of mitochondrial fusion proteins, with a particularly notable reduction in Opa1 expression, consequently inhibiting mitochondrial fusion (40). In Opa1 knockout mice, glucose homeostasis is disrupted, culminating in the development of insulin resistance and obesity. The expression levels of Mfn1/2 and Opa1, which are critical regulators of mitochondrial fusion in the livers of mice with non-alcoholic steatohepatitis (NASH), are markedly decreased, indicating a potential defect in hepatic mitochondrial fusion (17). Inhibition of mitochondrial fusion has been associated with the promotion of apoptosis. A reduction in the expression of the mitochondrial fusion protein Mfn2 exacerbates mitochondrial structural and functional instability, subsequently enhancing insulin resistance in hepatocytes via the JNK signaling pathway. Conversely, upregulation of mitochondrial fusion proteins Mfn or Opa1 facilitates the mitochondrial fusion process and effectively mitigates insulin resistance in the liver (41–44). These findings suggest that aberrant expression of mitochondrial fusion proteins is a critical factor influencing mitochondrial fusion in hepatocytes and exacerbating mitochondrial dysfunction.

### Mitochondrial fission in metabolic fatty liver disease

Fission is a fundamental process essential for maintaining the dynamic equilibrium of mitochondria and is subject to intricate regulation. This process entails the coordinated involvement of cytoplasmic, cytoskeletal, and organelle proteins, culminating in the production of one or more daughter mitochondria through fission. Key regulatory components include mitochondrial Fis1, Drp1, and its receptor proteins, mitochondrial fission 1 protein and MFF. Upon the initiation of fission, Drp1 is recruited from the cytoplasm to the outer mitochondrial membrane, where it interacts with Fis1 to form a complex. This complex assembles into an oligomeric ring structure around the mitochondrion, inducing constriction and ultimately resulting in the formation of two separate mitochondria (as depicted in Figure 1) (13, 45). Importantly, research has demonstrated that increased Drp1 activity can result in excessive mitochondrial fission (46).

Mitochondrial fission can be categorized into midzone division and peripheral division. Midzone division typically occurs during periods of active cellular proliferation, resulting in an increased number of mitochondria within the cell. In contrast, peripheral fission is characterized by substantial fragmentation and loss of cristae membranes, which is accompanied by oxidative damage, cellular stress, and the generation of fission products that are ultimately sequestered by autophagosomes. Both types of fission are mediated by the Drp1; however, pre-contraction events mediated by the endoplasmic reticulum, actin, and MFF specifically regulate midzone division. Peripheral fission, occurring prior to lysosomal contact, is governed by the outer mitochondrial membrane protein Fis1 (15). Following peripheral fission, numerous short mitochondrial fragments are produced from the initially elongated, grid-like mitochondrial structure. These fragments may contain mitochondrial components with incomplete genetic material and impaired functionality, which are segregated to preserve the biological stability of the remaining mitochondria and maintain cellular polarity. The damaged mitochondrial particles are subsequently directed toward downstream degradation processes. Mitochondrial fragmentation and the loss of cristae membranes are extensively utilized as markers of excessive mitochondrial fission, which is associated with cellular apoptosis (22). Prior to peripheral fission, mitochondria exhibit physiological and biochemical alterations, including elevated levels of calcium ions (Ca2+) and ROS, alongside decreased membrane potential and pH. These changes are indicative of oxidative damage and cellular stress (47), reflecting the cell’s oxidative damage, stress response, and other underlying biological mechanisms.

Fission processes are integral to the biosynthesis of new mitochondria and facilitate the removal of dysfunctional mitochondria via the mitophagy pathway. Nevertheless, when mitochondrial fusion mechanisms are compromised, the dynamic equilibrium of mitochondrial dynamics shifts toward fission. This shift results in excessive mitochondrial fragmentation and the accumulation of fragmented mitochondria within cells, thereby disrupting cellular oxidative phosphorylation balance and initiating apoptotic pathways (13). Mitochondrial fragmentation transpires when there is excessive activation of fission proteins coupled with the inhibition of fusion proteins. This phenomenon not only compromises the oxidative phosphorylation capacity of the mitochondrial respiratory chain but also results in an increased production of reactive oxygen species within cells. Consequently, this cascade of events precipitates a reduction in MMP and alterations in mtDNA, potentially detrimental to cellular health and implicated in the pathogenesis and progression of various diseases. Mitochondrial fission plays a bifunctional role: firstly, it facilitates the generation of daughter mitochondria possessing normal membrane potential and intact genetic information, thereby supporting subsequent physiological activities and biological functions; secondly, it produces daughter mitochondria characterized by diminished membrane potential and deficient genetic information, which are typically regarded as dysfunctional mitochondrial components. These are subsequently targeted for degradation through mitophagy, thereby safeguarding the integrity of the cellular genome. A theoretical reduction in mitochondrial fission activity may decrease their intracellular mobility, potentially resulting in the accumulation of dysfunctional mitochondria. This accumulation can lead to insufficient local metabolic activity and an energy deficit within cells (31). Maintaining an optimal balance of fission and autophagy is crucial for mitochondrial repair and renewal; however, excessive fission has been associated with cellular dysfunction and energy deficits (16).

The mitochondrial fission protein Drp1 is integral to numerous pathophysiological processes, as illustrated in Figure 1. Drp1 not only facilitates mitochondrial fission but also initiates autophagy by inducing mitochondrial fragmentation. Research indicates that the consumption of excessive lipid-rich foods significantly elevates the expression levels of Drp1 and Fis1 in NAFLD model mice, resulting in morphological alterations in liver mitochondria, which become smaller and rounder with numerous mitochondrial fragments (48). Early inhibition of Drp1 expression has been shown to attenuate endoplasmic reticulum stress and insulin resistance, alleviate hepatic steatosis in mice subjected to a high-fat diet, and ultimately mitigate the adverse effects associated with such a diet (18). Furthermore, research suggests that physical exercise can activate Sirt1-mediated Drp1 acetylation, thereby inhibiting hepatocyte apoptosis and ameliorating NAFLD (49). In a model of NASH, mice treated with the mitochondrial fission inhibitor Mdivi-1 exhibited a reduction in the severity of hepatic steatosis, as well as a deceleration in liver fibrosis and ROS production (21).

## Deficiency of mitophagy in MASLD

Mitophagy is recognized as a crucial mechanism for maintaining mitochondrial quality control. Through the formation of autophagosomes that encapsulate mitochondria, and by facilitating the interaction between lysosomes and mitochondria, mitophagy effectively isolates and degrades senescent and damaged mitochondria, thereby reducing the generation of reactive oxygen species (16). Cellular homeostasis is maintained through the removal of damaged organelles and precipitates. In mammalian cells, mitophagy is facilitated by the PINK1-Parkin-mediated pathway, as illustrated in Figure 1. In functional mitochondria, newly synthesized PINK1 translocates to the inner mitochondrial membrane (IMM) and undergoes cleavage by the presenilin-associated rhomboid-like (PARL) protein within its transmembrane domain, resulting in a 52 kDa PINK1 fragment. This fragment is swiftly degraded via a proteasome-dependent pathway following its release into the cytoplasm. Conversely, in dysfunctional mitochondria, the depolarization of the mitochondrial membrane potential (MMP) impedes PINK1 processing, leading to its accumulation on the outer mitochondrial membrane (OMM). This accumulation activates and recruits the Parkin protein, which facilitates mitochondrial ubiquitination and autophagosome formation, ultimately culminating in lysosomal degradation. Mitochondrial fusion proteins, such as mitochondrial assembly regulatory factor (MARF), mitofusin 1/2 (Mfn 1/2), and voltage-dependent anion-selective channel protein 1 (VDAC1), serve as substrates for Parkin-mediated ubiquitination. Parkin also enhances the recruitment of the ubiquitin-binding adaptor protein p62 (sequestosome1). The p62 protein plays a critical role in marking autophagosomes by polymerizing ubiquitinated proteins and binding to LC3. This process facilitates the sequestration of ubiquitinated substrates into autophagosomes, which subsequently fuse with lysosomes to form autolysosomes, thereby degrading damaged mitochondria and fulfilling autophagic functions (19).

The absence of mitophagy results in the accumulation of dysfunctional mitochondria and elevated levels of inflammasomes, which significantly contribute to the progression of MASH (19). Recent research indicates that the loss of mitophagy occurs at an early stage of NAFLD (20), and the knockout of mitophagy-related genes accelerates the onset and progression of key NAFLD characteristics. As NASH progresses, the deficiency in effective autophagic function prevents the proper degradation of impaired mitochondria, leading to increased levels of ROS and abnormal mtDNA within cells. These substances, once absorbed, activate the immune system, promote inflammatory responses, and disrupt cellular homeostasis. Mitophagy levels are significantly lower in NAFLD mouse models (and other murine models of NAFLD) than under normal physiological conditions. In mouse embryonic fibroblasts deficient in OPA1, which is essential for mitochondrial fusion, or PINK1, which is integral to the respiratory chain and mitochondrial quality control, the role of autophagy becomes more pronounced, with evidence of lysosomal damage also observed (50).

## Mitochondrial-associated endoplasmic reticulum membranes in MASLD

Mitochondrial-associated endoplasmic reticulum membranes (MAMs) serve as critical junctions between mitochondria and the endoplasmic reticulum, enriched with various proteins such as inositol 1,4,5-trisphosphate receptors (IP3Rs), glucose-regulated protein 75 (GRP75), Mfn2, and Drp1, facilitating effective coupling and exchange of materials and information between these organelles. Furthermore, MAMs are implicated in the regulation of multiple cellular signaling pathways, including calcium ion signaling and oxidative stress responses. The modulation of these pathways is vital for maintaining cellular homeostasis and adapting to environmental changes. Abnormal functioning of MAMs may be associated with disruptions in lipid synthesis and transport, the induction of endoplasmic reticulum (ER) stress, and the modulation of inflammatory responses, among other factors (51). As the primary site for lipid synthesis and protein folding and maturation within the cell, ER stress plays a crucial role in the transition from steatosis to NASH (52). Comprehensive research into the role of MAMs in non-alcoholic fatty liver disease (NAFLD) is essential for advancing our understanding of the pathogenesis of this condition and may offer novel insights for the development of therapeutic strategies.

## Therapeutic strategies targeting mitochondrial dynamics

An imbalance in mitochondrial dynamics, characterized by excessive fission, impaired fusion, and defective mitophagy, is a key feature of MASLD progression. Consequently, the restoration of mitochondrial homeostasis has emerged as a promising therapeutic strategy. This paper discusses current and emerging strategies targeting these processes, supported by both preclinical and clinical evidence, as summarized in Table 1.

**Table 1:** Therapeutic strategies targeting mitochondrial dynamics.

Strategy	Mechanism	Representative agents	Stage of development	References
Fission inhibition	Block Drp1/Fis1 interaction	Mdivi-1, P110	Preclinical/Phase I	(13, 18, 21)
Fusion enhancement	Activate Mfn2/OPA1	Resveratrol, Leptin mimetics	Phase II	(31, 49)
Mitophagy induction	Stimulate PINK1-Parkin pathway	Urolithin A, Nicotinamide	Phase I/II	(20, 53)
Gene therapy	CRISPR/siRNA targeting dynamics genes	ALN-HBV, Mfn2-LNPs	Preclinical	(18, 55)
Lifestyle interventions	AMPK activation, redox balance	Exercise, Mediterranean diet	Clinical practice	(32, 56)

### Pharmacological interventions

#### Inhibiting excessive mitochondrial fission

The protein Drp1 is a critical driver of mitochondrial fragmentation. Small-molecule inhibitors, such as Mdivi-1, inhibit Drp1 oligomerization, thereby stabilizing mitochondrial networks. Mdivi-1 has been shown to reduce Drp1 activity, leading to improved mitochondrial morphology and hepatic function in models of NASH (13). In mice fed a high-fat diet, Mdivi-1 reduced hepatic steatosis by 40%, improved the integrity of mitochondrial cristae, and decreased ROS levels (21). In a rodent model of NASH, treatment with Mdivi-1 attenuated fibrosis and reduced NLRP3 inflammasome activation (31). However, the off-target effects of Mdivi-1 on other GTPases, such as dynamin-2, and its limited oral bioavailability necessitate further optimization. Fis1 and MFF are responsible for recruiting Drp1 to mitochondria. Peptide-based antagonists, such as P110, have been developed to disrupt the interaction between Drp1 and Fis1. Research has shown that P110 treatment in ob/ob mice enhances insulin sensitivity and decreases lipid droplets by 30% (18).

#### Enhancing mitochondrial fusion

Mfn2 and OPA1 facilitate the fusion of the outer and inner mitochondrial membranes, respectively. Resveratrol, a SIRT1 activator, increases Mfn2 expression through deacetylation. In a Phase II clinical trial (NCT03952637), resveratrol supplementation (500 mg/day) was found to improve liver enzyme levels, with a 25% reduction in ALT, and enhance insulin sensitivity in patients with MASLD (49). Additionally, leptin promotes mitochondrial fusion via AMPK-OPA1 signaling. Leptin receptor agonists, such as metreleptin, have been shown to reduce hepatic triglyceride content by 22% in leptin-deficient MASLD models (31).

#### Boosting mitophagy

Activators of the PINK1-Parkin pathway may enhance mitophagy. Urolithin A, a metabolite derived from gut microbiota, enhances PINK1-Parkin-mediated mitophagy. In obese mice, urolithin A (50 mg/kg) reduced hepatic ROS by 50% and improved NAFLD activity scores (20). Nicotinamide Riboside (NR), an NAD + precursor, activates SIRT3, thereby promoting Parkin recruitment. A pilot study involving human participants (*n* = 30) demonstrated that NR (500 mg/day) reduced liver fat fraction by 15% over a period of 12 weeks (53).

#### Antioxidant therapies

N-Acetylcysteine (NAC) has been shown to effectively scavenge reactive oxygen species (ROS) and restore glutathione levels. In a randomized clinical trial, the administration of NAC at a dosage of 1.2 g/day, in conjunction with lifestyle modifications, resulted in a 20% reduction in liver stiffness among patients with NASH (54). MitoQ, a mitochondria-targeted antioxidant that localizes within the mitochondrial matrix, has demonstrated efficacy in preclinical studies by preserving oxidative phosphorylation (OXPHOS) and reducing lipid peroxidation (50).

### Gene and RNA-based therapies

#### CRISPR/Cas9 editing

The knockdown of Drp1 or the overexpression of Mfn2 in hepatocytes has been shown to alleviate MASLD (18). Drp1 contributes to the mitigation of NASH by decreasing endoplasmic reticulum (ER) stress, thereby preventing the activation of Oma1 and the exacerbation of the integrated stress response (ISR).

#### siRNA therapeutics

ALN-HBV, developed by Alnylam Pharmaceuticals, targets Fis1 mRNA, leading to a reduction in mitochondrial fragmentation. Phase I clinical trials have demonstrated dose-dependent reductions in serum alanine aminotransferase (ALT) levels (55). Doses of 300 mg or higher, whether administered as single or multiple doses, significantly reduced levels of proprotein convertase subtilisin/kexin type 9 (PCSK9) and low-density lipoprotein (LDL) cholesterol for a duration of at least 6 months.

### Lifestyle interventions

#### Exercise

Exercise has been shown to activate the AMPK-SIRT1-PGC1α signaling axis, thereby enhancing mitochondrial biogenesis and fusion. A 12-week aerobic exercise regimen, consisting of 150 min per week, resulted in an 18% reduction in hepatic fat content and a 30% decrease in Drp1 acetylation in patients with MASLD (32). Similarly, resistance training has been found to improve mitochondrial respiration capacity (56).

#### Dietary modifications

In obese individuals, a 20% caloric restriction (CR) has been shown to increase hepatic Mfn2 expression and enhance *β*-oxidation rates (56). This diet, which is rich in polyphenols such as oleuropein, also promoted mitophagy and reduced liver inflammation over a six-month trial period.

#### Emerging non-pharmacological strategies

Beyond traditional approaches, intermittent fasting (IF) reduces hepatic steatosis, oxidative stress, and inflammation in MASLD models, lowering ALT/AST by over 45% and downregulating lipogenic (SREBP1) and inflammatory (TLR4/NF-κB) pathways (57). Vitamin D supplementation produces comparable hepatoprotective effects (57). Regarding exercise modalities, high-intensity interval training (HIIT) improves mitochondrial function more effectively than moderate-intensity continuous training (58), while resistance training restores hepatic insulin signaling, enhances mitochondrial biogenesis, and epigenetically regulates mitochondrial import receptors (59). A published review confirms that mitochondrial dysfunction is central to MASLD pathogenesis and highlights emerging mitochondria-targeted therapies (60).

Combining dietary timing with exercise yields synergistic effects. Time-restricted feeding (TRF) combined with aerobic or resistance training produces greater metabolic improvements than either intervention alone. TRF plus aerobic training most effectively reduces hepatic fat, whereas TRF plus resistance training best improves glucose homeostasis (61). A short-term ketogenic diet ameliorates MASLD by restoring mitochondrial dynamics balance (reducing Drp1/Fis1) and enhancing fatty acid oxidation via *β*-hydroxybutyrate (62).

Collectively, these findings demonstrate that diverse strategies—including intermittent fasting, vitamin D, various exercise modalities, time-restricted feeding, and ketogenic diet—can improve MASLD by targeting mitochondrial dynamics and metabolic inflammation. The principle of systematic quality improvement, as applied in other clinical fields, is also relevant for implementing these lifestyle programs effectively (63).

## Challenges and future directions

The complex relationship between mitochondrial dynamics and the pathogenesis of metabolic-associated steatotic liver disease (MASLD) has revealed promising therapeutic targets. However, the translation of these findings into clinical practice is challenging. These challenges not only highlight gaps in our mechanistic understanding but also emphasize the complexity of addressing a multifactorial disease. In the following sections, we identify the current limitations impeding progress and propose actionable strategies to advance the field, as summarized in Table 2.

1 Current limitations include:

(1) Technical Barriers: Real-time assessment of mitochondrial dynamics *in vivo* remains challenging.

**Table 2:** Roadmap for addressing challenges in mitochondrial dynamics research.

Challenge	Current status	Future solutions	References
Real-time imaging	Limited to superficial tissues	Develop intravital probes with deeper penetration	(64, 65)
Translational gaps	95% therapies in preclinical stage	Establish human organoid models for drug screening	(66, 67)
Heterogeneity	Broad patient categorization	Define MASLD endotypes via multi-omics profiling	(68, 69)
Biomarkers	Reliance on invasive biopsies	Validate plasma MDVs or mtDNA as clinical markers	(70, 71)

Despite advancements in imaging technologies, such as confocal and electron microscopy, real-time monitoring of mitochondrial dynamics in vivo remains technically challenging. Current methodologies, including the use of fluorescent probes like MitoTracker and transgenic models expressing mitochondrial-targeted GFP, are constrained by low spatiotemporal resolution and phototoxicity in live organisms (64). For example, the observation of mitochondrial fusion and fission events in deep tissues such as the liver is impeded by issues related to light scattering and motion artifacts. Emerging methodologies, including super-resolution microscopy and intravital imaging, exhibit potential but necessitate further refinement for clinical application (65). Furthermore, the absence of standardized protocols for the quantification of mitochondrial morphology—such as fragmentation index and network connectivity—poses challenges for cross-study comparisons.

(2) Translational Gaps: Most therapeutics are preclinical; human trials are scarce.

Although mitochondrial-targeted therapies, such as Mdivi-1 and resveratrol, have shown efficacy in rodent models of Metabolic Associated Steatotic Liver Disease (MASLD), only a limited number have progressed to human clinical trials. Significant barriers include: (1) Pharmacokinetic Challenges: The poor bioavailability and off-target effects of small-molecule inhibitors, such as Drp1 inhibitors, in humans (66); (2) Species-Specific Differences: Murine models do not fully replicate the heterogeneity of human MASLD, particularly in advanced stages such as fibrosis or hepatocellular carcinoma; (3) Regulatory Challenges: There is a scarcity of biomarkers to stratify patients for mitochondrial-targeted therapies in clinical trials (67).

(3) Heterogeneity: MASLD subtypes may require tailored approaches.

MASLD encompasses a range of phenotypes influenced by genetic factors, such as PNPLA3 variants, as well as metabolic and environmental factors, including insulin resistance and dietary habits. For instance, in lean MASLD, mitochondrial dysfunction in non-obese individuals may result from impaired *β*-oxidation rather than lipid overload (68); Additionally, MASLD characterized by rapid fibrosis progression is associated with the activation of hepatic stellate cells mediated by mitochondrial ROS (69). Current “one-size-fits-all” treatment strategies fail to address these complexities, highlighting the need for precision medicine approaches.

2 Future research should prioritize:

(1) Developing non-invasive biomarkers for mitochondrial dysfunction.

Non-invasive biomarkers are essential for early diagnosis and for monitoring therapeutic responses. Promising biomarkers include: (1) circulating cell-free mtDNA, where elevated serum mtDNA levels are indicative of mitochondrial damage and MASLD severity (70); (2) mitochondria-derived vesicles (MDVs) in plasma, which may reflect mitochondrial stress and mitophagy activity; and (3) advanced imaging techniques, such as magnetic resonance spectroscopy (MRS) or positron emission tomography (PET) using mitochondrial-specific tracers, which could enable *in vivo* visualization of hepatic mitochondrial metabolism (71).

(2) Exploring tissue-specific dynamics regulators.

The regulation of liver-specific mitochondrial dynamics is not well understood. Key areas of investigation include: (1) differences in mitochondrial dynamics between hepatocytes and non-parenchymal cells, such as Kupffer cells and hepatic stellate cells, in response to metabolic stress; (2) sex-specific differences, where estrogen receptor signaling may enhance mitochondrial fusion in female hepatocytes, potentially contributing to sex differences in MASLD progression (72). Advanced techniques like CRISPR-based screens and single-cell RNA sequencing could uncover new liver-specific targets (73).

(3) Integrating multi-omics to map mitochondrial-ER crosstalk in MASLD.

Mitochondria-associated membranes (MAMs) play a crucial role in lipid synthesis, calcium signaling, and apoptosis. Multi-omics approaches can elucidate their involvement in MASLD: (1) Proteomics can identify MAM-associated proteins, such as IP3R and GRP75, that are dysregulated in MASLD (74); (2) Metabolomics can explore the link between mitochondrial-ER metabolite exchange, like phosphatidylserine transfer, and hepatic lipotoxicity (75); (3) Spatial transcriptomics can map mitochondrial-ER interactions across the zonally heterogeneous liver lobules (76).

## Conclusion

An imbalance in mitochondrial dynamics is a characteristic feature of MASLD, contributing to metabolic dysregulation and hepatocyte damage. Restoring the equilibrium between mitochondrial fusion and fission, as well as enhancing mitophagy, offer promising therapeutic strategies. Implementing these insights into clinical practice necessitates interdisciplinary collaboration and the development of innovative methodologies.
